# Supplementary material for: Reciprocal interactions between tumour cell populations enhance growth and reduce radiation sensitivity in prostate cancer
Source: Commun Biol. 2021 Jan 4;4:6. doi: 10.1038/s42003-020-01529-5 (PMC7782740; doi:10.1038/s42003-020-01529-5)
Supplement: Supplementary file 1 — Supplementary Information [file 42003_2020_1529_MOESM1_ESM.pdf]

SUPPLEMENTARY INFORMATION

Reciprocal interactions between tumour cell populations enhance growth and reduce radiation sensitivity in prostate cancer

Marcin Paczkowski, Warren W. Kretzschmar, Bostjan Markelc, Stanley K. Liu, Leoni A. Kunz-Schughart, Adrian L. Harris, Mike Partridge, Helen M. Byrne, Pavitra Kannan

SUPPLEMENTARY FIGURES

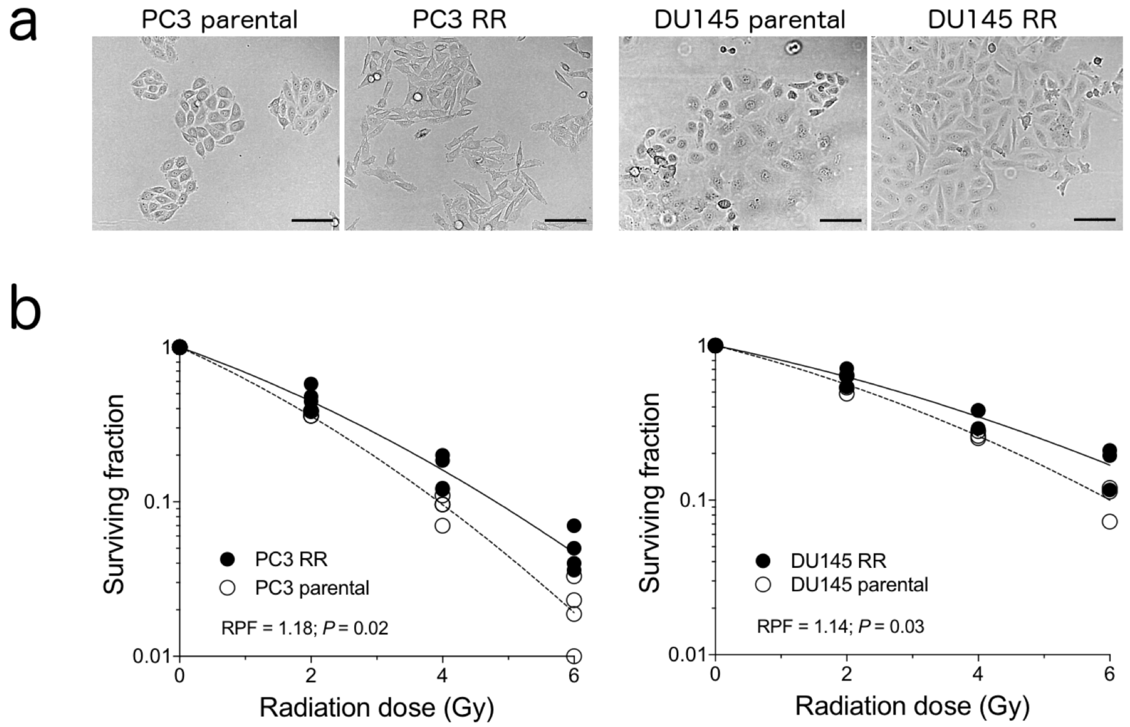

Supplementary Figure 1. Radioresistant (RR) populations from unlabelled PC3 and DU145 cell lines are morphological distinct from parental populations, and have enhanced clonogenic survival after radiation. **(a)** Morphology of parental and RR populations from PC3 and DU145 cell lines. **(b)** Clonogenic survival of PC3 (n = 4 experiments) and DU145 cells (n = 3 experiments) measured 10 days after irradiation with 0, 2, 4, and 6 Gy, and fitted to the linear-quadratic equation (the best fit is shown as a line connecting the mean of symbols). RPF = radiation protection factor. Significance was evaluated using Student's t-test (paired, one-tailed, α=0.05) on area-under-the-curve values.

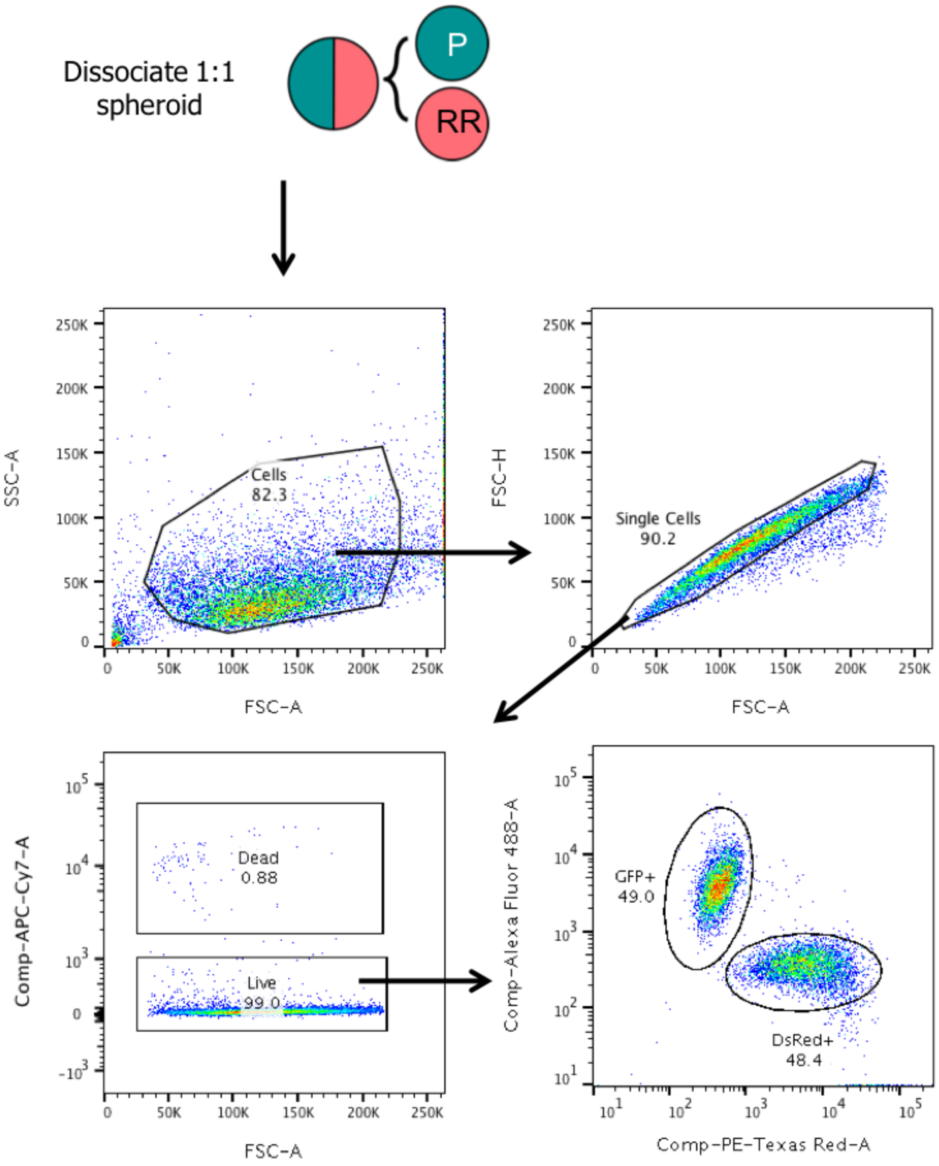

Supplementary Figure 2. Gating strategy for flow cytometry experiments measuring proportions of cell populations in spheroids. After spheroids (seeded 1:1 parental:RR) were dissociated, single cells were isolated from debris (SSC-A vs FSC-A) and doublets (FSC-H vs FSC-A). After gating of live and dead cells (efluor 780; Apc-Cy7-A vs FSC-A), parental (GFP+) and RR (DsRed+) cells were identified (Alexa Fluor 488-A vs PE-Texas Red-A). Percentages shown are derived from the proportion of cells of the previous gate. A total of 10,000 events were collected. SSC-A, side-scatter area; FSC-A, forward-scatter area; FSC-H, forward-scatter height.

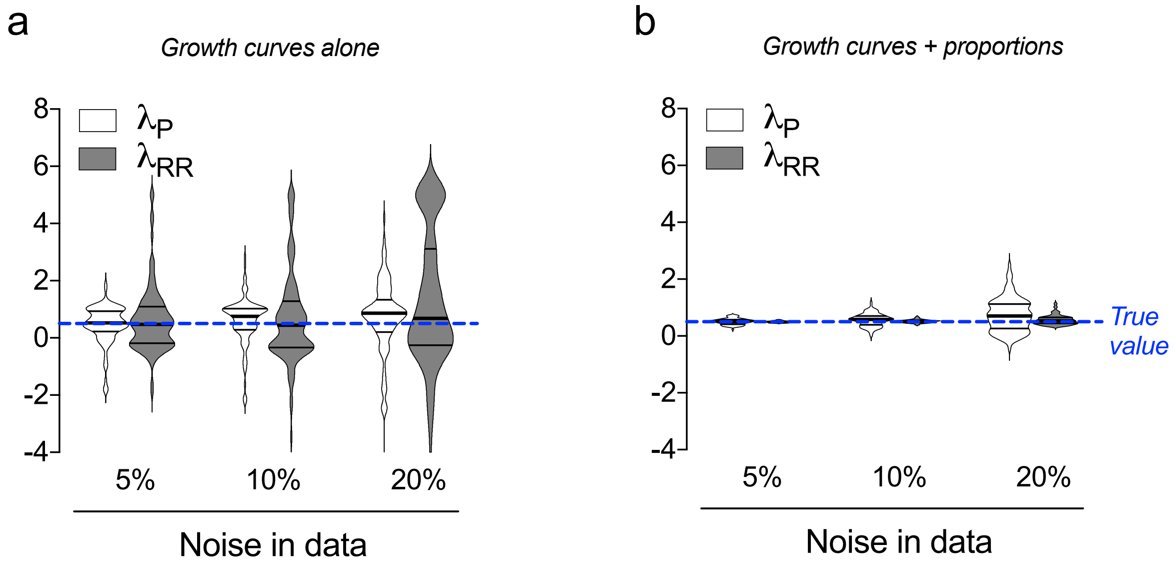

Supplementary Figure 3. Interaction parameters can be estimated mathematically using spheroid growth curves and proportions of cell populations. **(a)** Values of interaction parameters, λ_P and λ_{RR} (day^{-1} mm^{-1}), estimated from 100 simulated growth curves of mixed populations comprising 1:1 parental:RR populations. Growth curves were generated with different levels of noise to simulate biological variance. **(b)** Values of interaction parameters estimated using 100 simulated growth curves supplemented with experimental data on the proportion of each population. Data from all simulations are plotted as kernel density estimates (violin plot), with lines indicating median and quartiles.

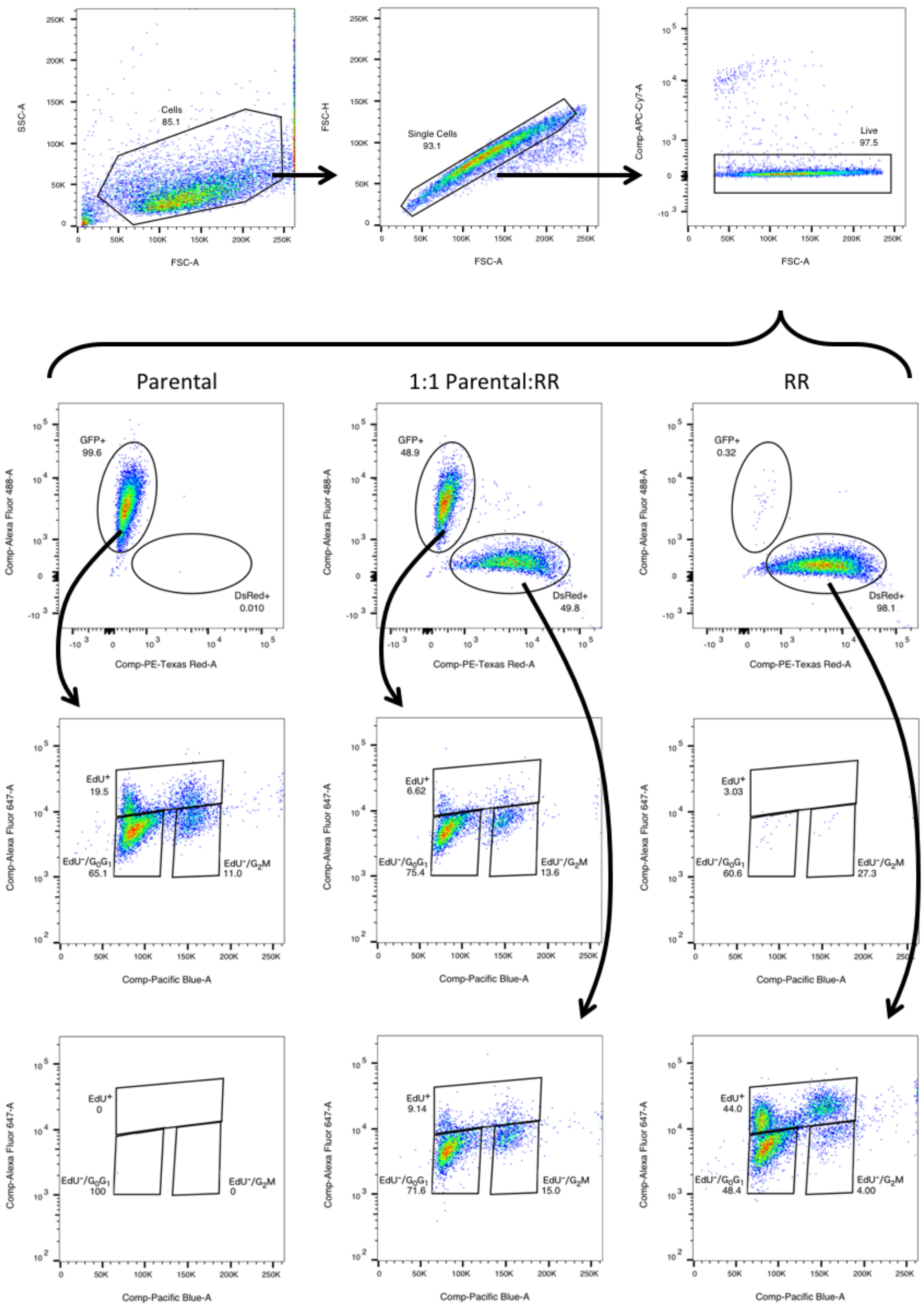

Supplementary Figure 4. Gating strategy for flow cytometry experiments measuring cell cycle for each cell population in homogeneous and mixed spheroids. After excluding debris (top left), doublets (top middle), and dead cells (top right, Cy7), parental (GFP+, Alexa Fluor 488) and RR (DsRed+, PE-Texas Red) cells were analysed for cell cycle proportions. The uptake of the nucleoside analogue 5-ethynyl-2 deoxyuridine (EdU, Alexa Fluor 647) was detected both in cells proliferating in S phase and in cells that previously took up EdU in S phase and then cycled into G_0G_1 (Pacific Blue) during the incubation time. Gating was thus performed to include all proliferation (EdU+ cells) to account for turnover.

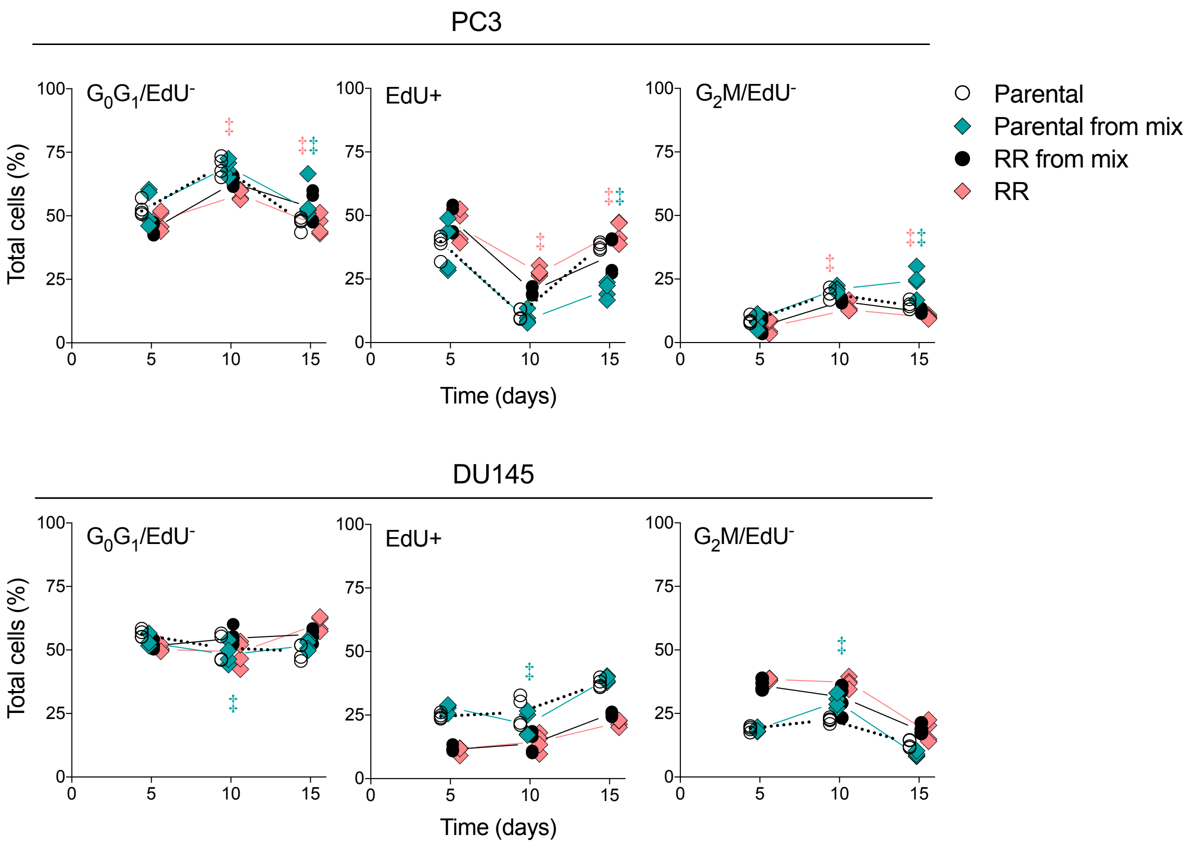

Supplementary Figure 5. Altered cell cycle turnover in mixed PC3 and DU145 spheroids after 6 Gy radiation, as assessed by flow cytometry. Cell cycle phases for parental and RR cell populations isolated from either mixed or homogeneous spheroids. For all data shown, n = 4 experiments; lines connecting symbols indicate median value. ‡*FDR* < 0.05, overdispersed binomial regression with Benjamini-Hochberg correction.

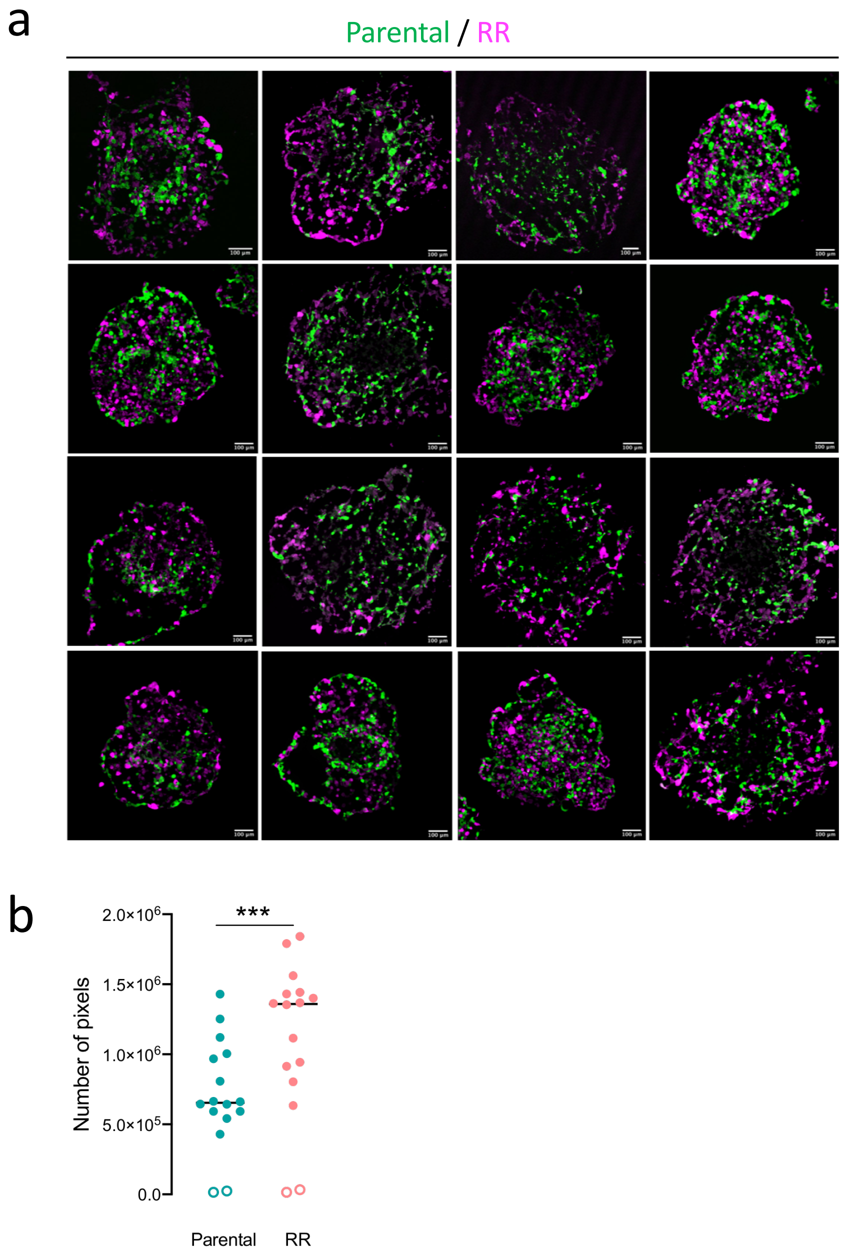

Supplementary Figure 6. (a) Images showing spatial localisation of parental (green pseudo-colour) and RR (magenta pseudo-colour) cell populations in 16 different mixed PC3 spheroids isolated on day 11. To check the reproducibility of the localisation, spheroids were obtained from 4 different batches and, as a result, were imaged at different times. Individual colour channels were adjusted using homogenous spheroids (controls) imaged in each batch. Scale bar = 100 µm. **(b)** Quantification of pixels (5 * median of the background) from parental (green circles) and RR (pink circles) populations in each spheroid cross-section (n = 16 spheroids). The four points shown in open circles are paired values from two spheroids that were imaged with a lower bit-resolution (8 instead of 16), resulting in lower pixel values. ***P <0.001 by two-tailed, Wilcoxon matched-pairs signed rank test. Line indicates the median value.

Supplementary Methods

Supplementary Methods 1: Spatially-averaged mathematical models

Logistic model of homogeneous spheroid growth

The growth of tumour spheroids typically begins with a phase of exponential growth which is followed by an intermediate linear growth phase and eventually superseded by a slower approach to an equilibrium size at which the net rates of cell death and proliferation across the tumour volume are balanced. Growth laws that have been proposed to describe this process include the Gompertz, Bertalanffy and logistic models [5]. Here we use the latter to model the growth of homogeneous spheroids. The logistic growth law states that the rate of change of the tumour spheroid volume $V(t)$ (mm^3) at time t (day) is given by

$$\frac{dV}{dt} = rV\left(1 - \frac{V}{K}\right), \text{ with } V(t=0) = V_0, \tag{1}$$

where the parameters $r > 0$ (day^{-1}) and $K > 0$ (mm^3) represent the growth rate and carrying capacity, or equilibrium size, of the tumour and $V_0 > 0$ (mm^3) denotes the spheroid volume at $t = 0$. The initial value problem (1) can be solved to give

$$V(t) = \frac{V_0 K e^{rt}}{K + V_0(e^{rt} - 1)}.$$

Lotka-Volterra model of heterogeneous spheroid growth

We assume that when the control and resistant cell populations are co-cultured to form heterogeneous spheroids, their growth dynamics and interactions can be viewed as a Lotka-Volterra system. If we denote by $V_C(t)$ and $V_R(t)$ the volumes of the tumour occupied by the control and resistant cells respectively at

time t, then their evolution is described by the following system of ordinary differential equations:

$$\begin{aligned} \frac{dV_C}{dt} &= V_C \left[r_C \left(1 - \frac{V_C}{K_C} \right) - \lambda_R V_R \right], \\ \frac{dV_R}{dt} &= V_R \left[r_R \left(1 - \frac{V_R}{K_R} \right) - \lambda_C V_C \right]. \end{aligned} \tag{2}$$

In Eqs. (2), the positive parameters r_C (day^{-1}) and r_R (day^{-1}) are the intrinsic growth rates of the two populations and the positive parameters K_C (mm^3) and K_R (mm^3) their carrying capacities (i.e., the equilibrium volumes to which the control and resistant populations would evolve if they were cultured as homogeneous spheroids). The parameters λ_C (day^{-1} mm^{-1}) and λ_R (day^{-1} mm^{-1}) respectively describe the effect that the control cells have on the resistant cells, and vice versa. In the absence of prior knowledge about the nature of the interactions between the two cell populations, we do not restrict λ_C and λ_R to be positive or negative. Indeed, as the signs of λ_C and λ_R vary we can distinguish six types of interactions: these are summarised in Table 1. We note that, if $\lambda_C = \lambda_R = 0$, then Eqs. (2) reduce to two logistic

Table 1: Potential interactions between tumour cell populations. The table summarises the biological effects that the control and resistant cell populations can have on each other, together with the corresponding signs of the parameters λ_C and λ_R in Eqs. (2).

**Type of interaction**	**Effect of control cells on resistant cells**	**Effect of resistant cells on control cells**	**Sign of λ_C**	**Sign of λ_R**
Competition	Detrimental	Detrimental	$\lambda_C > 0$	$\lambda_R > 0$
Amensalism	Detrimental	No effect	$\lambda_C > 0$	$\lambda_R = 0$
Antagonism	Detrimental	Beneficial	$\lambda_C > 0$	$\lambda_R < 0$
Neutralism	No effect	No effect	$\lambda_C = 0$	$\lambda_R = 0$
Commensalism	No effect	Beneficial	$\lambda_C = 0$	$\lambda_R < 0$
Mutualism	Beneficial	Beneficial	$\lambda_C < 0$	$\lambda_R < 0$

equations for V_C and V_R similar to Eq. (1).

In order to arrive at a well defined initial value problem, Eqs. (2) are supplemented by the following initial conditions:

$$V_C(t = 0) = V_{C0} \qquad \text{and} \qquad V_R(t = 0) = V_{R0}. \tag{3}$$

For a homogeneous spheroid, whose growth can be modelled using Eq. (1), there is an obvious physical interpretation of the carrying capacity parameter K – it is the maximum volume of the spheroid that can be supported by its environment. For a heterogeneous spheroid, such as that modelled with Eqs. (2),

the spheroid's saturation size cannot be described by a single parameter. Indeed the different interactions that may exist between co-cultured cell populations manifest themselves in a multitude of possible growth regimes. Nonetheless, we can consider some simple cases. Let us first denote by K_T the carrying capacity of a heterogeneous spheroid, when it exists. If $\lambda_C = \lambda_R = 0$ then it is straightforward to show that $K_T = K_C + K_R$. In the case of competition, when $\lambda_C > 0$ and $\lambda_R > 0$, we would expect $K_T < K_C + K_R$. Similarly, when the populations support each other, that is when $\lambda_C < 0$ and $\lambda_R < 0$ (mutualism), we anticipate that $K_T > K_C + K_R$.

For logistic growth, the carrying capacity K corresponds to a stable steady state (see Eq. (1)). Similarly, the co-existence equilibrium solutions of Eqs. (2), where they exist and are stable, define the saturation sizes of heterogeneous spheroids. By setting time-derivatives equal to zero in Eqs. (2), it is straightforward to show that Eqs. (2) possess up to four steady states:

1. $(V_C, V_R) = (0, 0)$: tumour elimination;
2. $(V_C, V_R) = (K_C, 0)$: homogeneous tumour spheroid, with radio-resistant cells eliminated;
3. $(V_C, V_R) = (0, K_R)$: homogeneous tumour spheroid, with radio-sensitive cells eliminated;
4. $(V_C, V_R) = (V_C^*, V_R^*)$: coexistence of both cell populations, where

$$V_C^* = \frac{\left(\frac{1}{K_R} - \frac{\lambda_R}{r_C}\right)}{\left(\frac{1}{K_R K_C} - \frac{\lambda_R \lambda_C}{r_R r_C}\right)} \quad \text{and} \quad V_R^* = \frac{\left(\frac{1}{K_C} - \frac{\lambda_C}{r_R}\right)}{\left(\frac{1}{K_R K_C} - \frac{\lambda_R \lambda_C}{r_R r_C}\right)},$$

$$\Rightarrow V_T = V_C^* + V_R^* = \frac{\left(\frac{1}{K_R} + \frac{1}{K_C}\right) - \left(\frac{\lambda_R}{r_C} + \frac{\lambda_C}{r_R}\right)}{\left(\frac{1}{K_R K_C} - \frac{\lambda_R \lambda_C}{r_R r_C}\right)}.$$

Steady states 1, 2 and 3 exist for all choices of the interaction parameters λ_C and λ_R. By contract, the coexistence steady state is only physically realistic and stable for certain combinations of values of r_C, r_R, K_C, K_R, λ_C and λ_R. In particular, if we define $\eta_R = \frac{\lambda_R K_R}{r_C}$ and $\eta_C = \frac{\lambda_C K_C}{r_R}$ then the coexistence steady state exists in the shaded regions of Fig. 1 where

$$V_C^* = \frac{(1 - \eta_R)}{(1 - \eta_R \eta_C)} K_C \quad \text{and} \quad V_R^* = \frac{(1 - \eta_C)}{(1 - \eta_R \eta_C)} K_R,$$

$$\Rightarrow V_T = \frac{(1 - \eta_R) K_C + (1 - \eta_C) K_R}{(1 - \eta_R \eta_C)}.$$

We note also that if $\eta_R = \eta_C = \eta$, say, then $V_T = (1 + \eta)(K_C + K_R)$, in which case $0 < V_T < (K_C + K_R)$ for $-1 < \eta < 0$ and $V_T > (K_C + K_R)$ for $0 < \eta$.

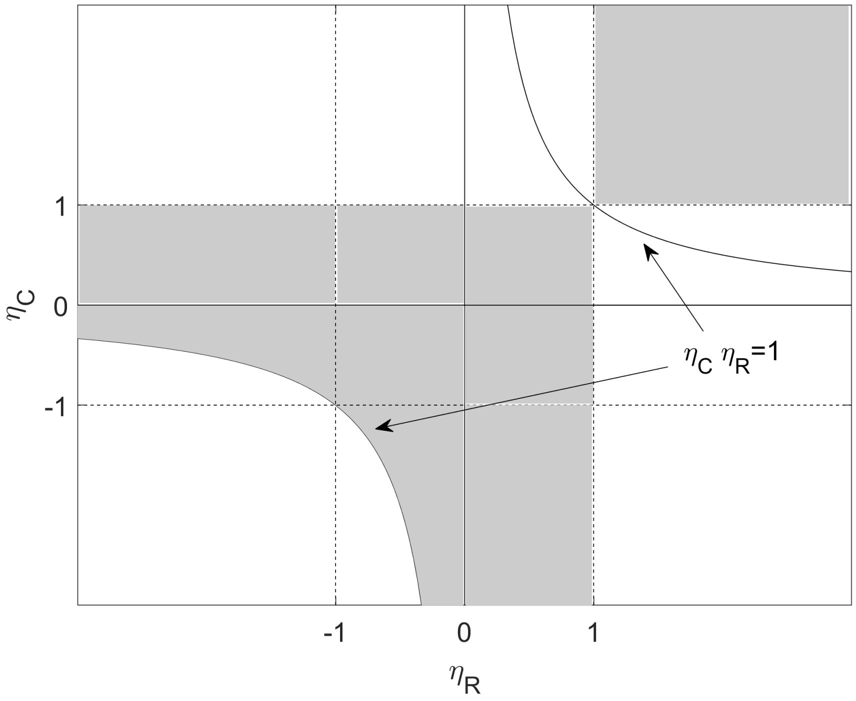

Figure 1: The coexistence steady state is physically realistic and stable in the shaded regions of the $\eta_R\eta_C$-plane, where $\eta_R = \frac{\lambda_R K_R}{r_C}$ and $\eta_C = \frac{\lambda_C K_C}{r_R}$.

Parameter estimation

Logistic model

The values of parameters $\theta_{hom} = (r, K, V_0)$ in Eq. (1) were estimated by minimising the weighted sum of squared residuals between experimental measurements of spheroid volume and the solution to Eq. (1) for given values of θ_{hom}. In mathematical terms, we sought $\hat{\theta}_{hom}$ such that

$$\hat{\theta}_{hom} = \arg\min_{\theta_{hom}} \sum_{i=1}^{N} \left[\frac{V_i^{data} - V^{model}(t_i;\theta_{hom})}{V^{model}(t_i;\theta_{hom})} \right]^2 \tag{4}$$

where V_i^{data} are the volume measurements taken at times t_i ($i = 6$ for PC3 and $i = 9$ for DU145 spheroids, see Fig. 1b in the main text) and $V^{model}(t_i;\theta_{hom})$ are model outputs at t_i for given values of the model parameters θ_{hom}.

We remark that there is no consensus regarding how to weight the data when minimising the sum of squared residuals. In equation (4), we follow [1] and use the model to weight the data.

Lotka-Volterra model

The values of the parameters $r_C, r_R, K_C, K_R, V_{C0}$ and V_{R0} describing the growth rates, carrying capacities and initial volumes for each cell population were estimated using the volume data collected from the homogeneous spheroids, and were subsequently fixed in Eqs. (2). Thus the only unknown parameters in Eqs. (2) were the interaction parameters $\theta_{het} = (\lambda_C, \lambda_R)$. These were estimated by seeking θ_{het} that minimises the following sum of squared residuals:

$$\sum_{j=1}^{M}\left[\frac{V_C^{data}(t_j) - V_C^{model}(t_j;\theta_{het})}{V_C^{model}(t_j;\theta_{het})}\right]^2 + \sum_{j=1}^{M}\left[\frac{V_R^{data}(t_j) - V_R^{model}(t_j;\theta_{het})}{V_R^{model}(t_j;\theta_{het})}\right]^2 + \sum_{i=1}^{N}\left[\frac{V_T^{data}(t_i) - V_T^{model}(t_i;\theta_{het})}{V_T^{model}(t_i;\theta_{het})}\right]^2, \tag{5}$$

where $V_C^{data}(t_j)$ and $V_R^{data}(t_j)$ respectively represent the volumes of the control and resistant populations within the heterogeneous spheroids at times t_j, $V_C^{model}(t_j;\theta_{het})$ and $V_R^{model}(t_j;\theta_{het})$ are solutions to Eqs. (2) at t_j for given values of θ_{het}, $V_T^{data}(t_i) = V_C^{data}(t_i) + V_R^{data}(t_i)$ are the total volumes of the heterogeneous spheroids at times t_i and $V_T^{model}(t_i;\theta_{het}) = V_C^{model}(t_i;\theta_{het}) + V_R^{model}(t_i;\theta_{het})$ are the corresponding solutions to Eqs. (2) at t_i for θ_{het}. The times t_i at which total spheroid volumes were measured are shown in Fig. 1b (main text). The times t_j at which the proportions of the control and resistant cells were measured are $t_j = (5, 10, 15, 19)$ (days) for PC3 and $t_j = (5, 10, 15)$ (days) for DU145 spheroids.

The minimisation problems (4) and (5) were implemented and solved in MATLAB using a non-linear least squares solver *lsqnonlin* which is an implementation of the trust-region-reflective iterative optimisation algorithm [3]. Since *lsqnonlin* is a local solver we use it in combination with *MultiStart*, a function that runs the local solver from a number of randomly selected starting points within a prescribed region of parameter space, thus ensuring more thorough exploration of the parameter space.

Radiation response modelling for theoretical study

To simulate growth of heterogeneous spheroids after exposure to radiation we used Eqs. (2) for a range of values of λ_C and λ_R (with other parameter values fixed) to grow *in silico* spheroids until they reached a volume of $V_{IR} = 0.9\text{mm}^3$. We then simulated radiation damage by calculating the surviving fraction σ of each population according to the linear-quadratic model [9]. In particular we set

$$V_\gamma(t_{IR^+}) = \sigma_\gamma V_\gamma(t_{IR^-}) \tag{6}$$

where

$$\sigma_\gamma = e^{-(\alpha_\gamma d + \beta_\gamma d^2)} \tag{7}$$

for $\gamma \in \{C, R\}$. In Eqs. (6) and (7) $t_{IR^\pm}$ represent the times just before (t_{IR^+}) and just after (t_{IR^-}) irradiation, and α_γ (Gy^{-1}) and β_γ (Gy^{-2}) are the lethal lesions made by one- and two-track actions of radiation dose d. Following radiation we used Eqs. (2) to regrow the *in silico* spheroids until they reached their pre-irradiation volume of 0.9mm^3. Values of the parameters α_γ and β_γ were estimated for each cell population using data collected from the clonogenic assays. The resulting growth curves were used to generate surface plots representing the regrowth time (Figure 4 in the main text).

Supplementary Methods 2: Spatially resolved computational model

We developed a 2D hybrid cellular automaton model of avascular tumor growth and assumed that it is representative of the changes in the size and structure of a 2D cross-section through a 3D tumor spheroid suspended in culture medium. The hybrid CA model couples a set of automaton elements, each with size $l \times l$, arranged on a regular $L \times L$ grid to a reaction-diffusion equation (RDE) describing the spatial distribution of a growth-rate-limiting nutrient which is supplied from the culture medium surrounding the spheroid. Unless otherwise stated, we fix $L = 200$ and take the size of each automaton element to represent the size of an average PC3 cell ($l = 18$ µm). Then the grid represents a region of size 0.36×0.36 cm^2. We consider oxygen (O_2) to be the growth-rate-limiting nutrient and model its concentration explicitly. The reaction-diffusion equation is discretised and solved on the same 2D grid as the cellular automaton (see Section for details). Each automaton element is occupied either by a cell or by culture medium and has associated with it an oxygen concentration. The behaviour of cells on the grid depends on their local O_2 concentration and the occupancy of their neighbourhood. These factors determine the rate at which cells consume O_2, and whether they proliferate, become quiescent or die.

In the remainder of this section we describe how the reaction-diffusion equation was discretised and solved, outline the rules governing our CA model and summarise the parameters (and parameter values) used in the model simulations.

Oxygen dynamics

A reaction-diffusion equation models the concentration of oxygen within our simulation domain. If we denote the oxygen concentration at location $\mathbf{x}$ and time t by $c(\mathbf{x}, t)$ (mol cm^{-3}) then its evolution is described by

$$\frac{\partial c(\mathbf{x}, t)}{\partial t} = D\nabla^2 c(\mathbf{x}, t) - \Gamma(\mathbf{x}, t), \tag{8}$$

where D is the (constant) oxygen diffusion coefficient ($\text{cm}^2\ \text{s}^{-1}$) and $\Gamma(\mathbf{x}, t)$ is the oxygen consumption rate ($\text{mol cm}^{-3}\ \text{s}^{-1}$). In what follows, we fix

$$\Gamma(\mathbf{x}, t) = \begin{cases} \kappa_{\mathcal{P}_\gamma} & \text{if } \mathbf{x} \text{ is occupied by a proliferating cell from population } \gamma, \\ \kappa_{\mathcal{Q}_\gamma} & \text{if } \mathbf{x} \text{ is occupied by a quiescent cell from population } \gamma, \\ 0 & \text{otherwise,} \end{cases} \tag{9}$$

where $0 < \kappa_{\mathcal{Q}} < \kappa_{\mathcal{P}}$ denote respectively the constant rates at which quiescent and proliferating cells consume oxygen (units: moles $\text{cm}^{-3}\ \text{s}^{-1}$). Equation (8) is supplemented by the following initial and boundary conditions

$$c(x, y, 0) = c_\infty, \tag{10}$$

$$c(0, y, t) = c(L, y, t) = c(x, 0, t) = c(x, L, t) = c_\infty \tag{11}$$

where L is the domain length and c_∞ is the background O_2 concentration. Equations (8)–(11) describe a situation in which O_2 diffuses from the boundaries of a (rectangular) Petri dish (where it is maintained at fixed levels) into the culture medium where it is consumed by tumour cells. Although in theory the O_2 concentration could become negative, in practice this does not happen because cells residing in low, but nonzero, O_2 conditions become necrotic and do not consume O_2.

Eqs. (8)–(11) are nondimensionalised, discretised using central differences for the spatial derivatives and forward differences for the time derivatives, and then solved using an explicit Euler scheme in MATLAB.

Cellular automaton rules

Cell status

Each automaton element at location $\mathbf{x} = (x, y)$ and time t can be considered a dynamical variable with a *state* and *neighbourhood.* Possible *states* include proliferating ($\mathcal{P}_\gamma$), quiescent ($\mathcal{Q}_\gamma$), necrotic ($\mathcal{N}_\gamma$) and empty ($\mathcal{E}$) cells (empty automaton elements are assumed to contain culture medium) which enables us to represent commonly observed features of tumour spheroids which include the formation of quiescent/hypoxic and necrotic regions in response to oxygen levels. We define quiescent cells to be viable cells that are not actively progressing through the cell cycle. These cells are in the G_0 phase and awaiting restoration of favourable conditions so that they can re-enter the cell cycle. This CA can be extended to model the growth of heterogeneous spheroids by introducing more states. For example, by defining $\gamma \in (\mathcal{C}, \mathcal{R})$ *states* becomes $\{\mathcal{P}_\mathcal{C}, \mathcal{P}_\mathcal{R}, \mathcal{Q}_\mathcal{C}, \mathcal{Q}_\mathcal{R}, \mathcal{N}_\mathcal{C}, \mathcal{N}_\mathcal{R}, \mathcal{E}\}$ allowing us to model the growth of heterogeneous spheroids consisting of control ($\mathcal{C}$) and resistant ($\mathcal{R}$) cells.

The *state* of a cell at location $\mathbf{x}$ and time t, denoted as $state(\mathbf{x}, t)$, is determined by the concentration of oxygen, $c(\mathbf{x}, t)$, at that location. If we define by $c_{\mathcal{Q}\gamma}$ and $c_{\mathcal{N}\gamma}$ the threshold oxygen levels below which cells of type γ become quiescent and necrotic, respectively, then we have:

- if $c_\infty \geq c(\mathbf{x}, t) > c_{\mathcal{Q}\gamma}$ then $state(\mathbf{x}, t) = \mathcal{P}_\gamma$,
- if $c_{\mathcal{Q}_\gamma} \geq c(\mathbf{x}, t) > c_{\mathcal{N}_\gamma}$ then $state(\mathbf{x}, t) = \mathcal{Q}_\gamma$,
- if $c_{\mathcal{N}_\gamma} \geq c(\mathbf{x}, t) \geq 0$ then $state(\mathbf{x}, t) = \mathcal{N}_\gamma$.

In addition to a *state*, every automaton element has a *neighbourhood* associated with it. The *neighbourhood* provides local information about the cell's surroundings and can impact the cell's next *state*. The two most commonly used neighbourhoods in CA models are the von Neumann and Moore neighbourhoods (Fig. 2). The number and location of neighbours can have a significant impact on cell-cell communication. Since it has been previously demonstrated that the Moore neighbourhood can minimise artefacts associated with lattice anisotropies [10] we use the first order Moore neighbourhood. Thus, for a 2D model, every cell communicates with its eight nearest neighbours.

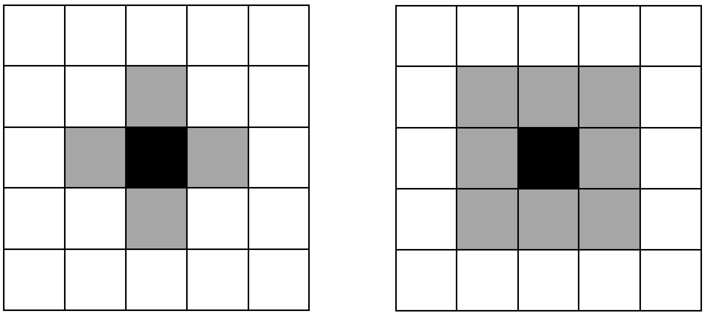

(a) First order von Neumann neighbourhood

(b) First order Moore neighbourhood

Figure 2: Two neighbourhoods that are commonly used in CA models.

Cell cycle progression

Every automaton element occupied by a proliferating cell is assigned a *counter* that monitors its cell cycle progression. When a new cell is created following cell proliferation, its *counter* τ_{cycle_γ} is a random number drawn from a normal distribution with mean $\bar{\tau}_{cycle_\gamma}$ and standard deviation σ_{cycle_γ} where $\bar{\tau}_{cycle_\gamma}$ represents the average cell cycle duration for cell population γ. After each discrete time step τ, the cell cycle counter τ_{cycle_γ} for a given cell is reduced by an amount that depends on its local *neighbourhood* (see Fig. 3). This

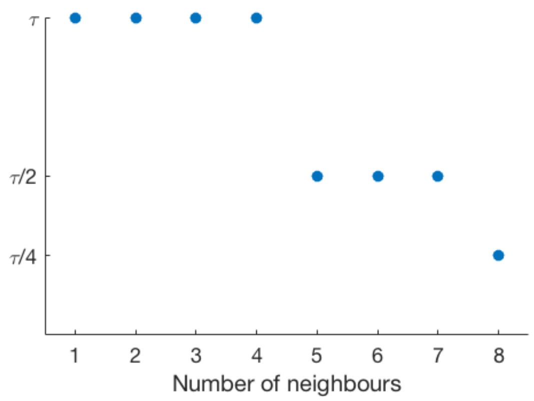

Figure 3: The amount by which τ_{cycle_γ} for a given cell is reduced after each computational step of length τ as a function of the number of the cell's neighbours.

mechanism imitates contact inhibition of proliferation, a well known feature of 2D and 3D cell aggregates. Unlike Jagiella et al. [7] who assumed that a cell cycles only if it is located within a given distance from the spheroid boundary, here a cell makes this decision based on local information only.

Cell division

When $\tau_{cycle_\gamma} \leq 0$ for a given cell, it divides to produce two identical cells. One cell occupies the same position as its parent and the other cell is placed in an adjacent automaton element. If more than one automaton element adjacent to the dividing cell is empty then the division process is complete (if a dividing cell has more than one free neighbour then the neighbour with the maximum number of neighbours is chosen to maintain cell-cell adhesion [8]). Alternatively, if a dividing cell does not have an empty adjacent automaton element, then we find the shortest chain of cells that connects the dividing cell to the spheroid's boundary. We shift the cells in the chain along this path in order to create space for the new daughter cell (if multiple shortest paths exist we choose one at random). This algorithm mimics the way in which growing cells exert mechanical stress on their neighbours to generate spheroid expansion. Fig. 4 illustrates how the chain of cells 1, 2 and 3 is shifted towards the spheroid's boundary to create space for the new daughter cell D.

Cell death

A cell becomes necrotic if the oxygen concentration at its location falls below a threshold value $c_{\mathcal{N}_\gamma}$. Necrotic cells are lysed at the rate p_{lys_γ} (h^{-1}). When a necrotic cell is lysed it is removed from the computational grid and a chain of cells is shifted from the spheroid's boundary towards the location of the removed cell

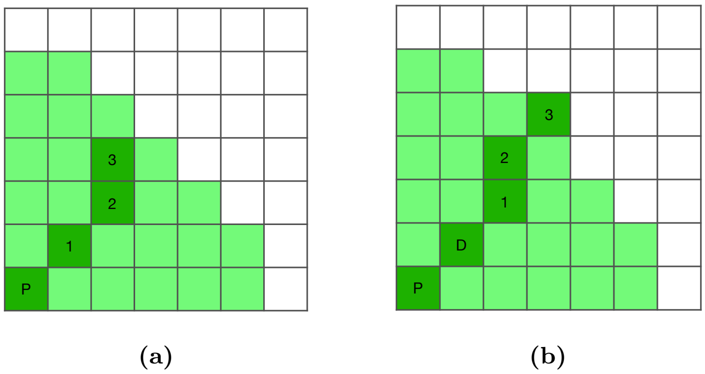

Figure 4: Chain shifting following a cell division event. State of the grid (a) before and (b) after division. The dividing cell P pushes the chain of cells 1, 2 and 3 toward the spheroid's boundary to create space for the new daughter cell D. Key: proliferating cells (green); empty cells (white); chain of shifted cells (dark green).

(see Fig. 5). In order to preserve spheroidicity we select the cell on the boundary that is located furthest away from the spheroid center (or choose randomly if multiple cells lie at the same distance from the center). The resulting cell rearrangement occurs within one computational time step and ensures that the spheroid remains compact.

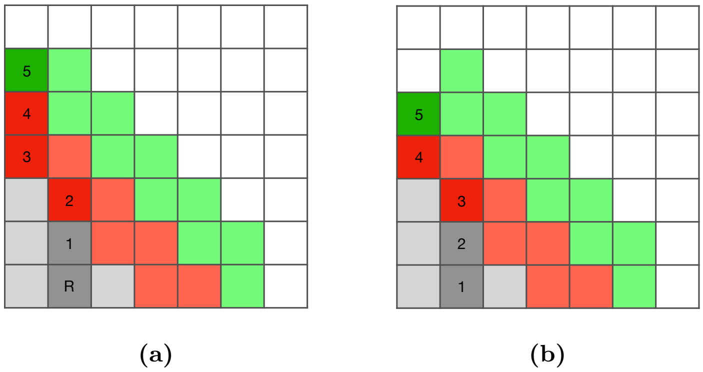

Figure 5: Chain shifting following lysis. State of the grid (a) before and (b) after cell removal. A chain of cells 1, 2, 3, 4 and 5 are shifted following lysis of cell R. Key: proliferating cells (green); quiescent cells (red); necrotic cells (grey); empty cells (white).

Algorithm summary

We now outline the computer algorithm used to implement the hybrid CA model.

1. *Initialisation* ($t = 0$). We assign values to all model parameters (see Table 2), initialise the oxygen distribution on the spatial grid, and place a cluster of proliferating control and resistant cells at the

centre of the computational grid (all other grid sites are assumed to contain culture medium), assign cell cycle duration times to the cells and set $t = \tau$ where τ is the length of our computational step.

2. *Nutrient consumption.* We update the oxygen concentration by solving Eqs. (8)–(11).

3. *Update cell states.* We update the states of all cells based on the updated oxygen distribution (see Sec.).

4. *Update cell cycle times.* The cell cycle duration *counter* of each proliferating cell is decreased by an amount that depends on the number of cells in its first order Moore neighbourhood (see Fig. 3).

5. *Check for cell division.* If the updated cell cycle *counter* of a proliferating cell satisfies $\tau_{cycle} \leq 0$ then the cell divides. Cell division is performed asynchronously (i.e., we randomly loop over all cells marked for division).

6. *Check for lysis of necrotic cells.* Necrotic cells are removed from the grid at rate p_{lys}. Cell lysis is performed asynchronously.

7. *Update time.* Time t is increased by a computational step τ. If $t < T$, where T is the total simulation time, then steps (2)–(7) are repeated; if $t \geq T$, then the simulation ends.

Our hybrid CA model was implemented in MATLAB. A flowchart summarising the algorithm is presented in Fig. 6.

Hybrid CA model parameter estimates

Oxygen concentration

The background oxygen concentration was set to $c_\infty = 2.8 \times 10^{-7}$ mol cm^{-3} [4] and the oxygen diffusion constant within a growing spheroid was set to $D = 1.8 \times 10^{-5}$ cm^2 s^{-1} [6]. Since the diffusion of O_2 in culture medium is likely to be higher than within the spheroid [7], we assumed that the concentration of O_2 within the medium was replenished on a faster timescale than within the spheroid. In practice this means that O_2 levels in the culture medium were held constant at the background O_2 concentration c_∞. Proliferating cells were assumed to consume oxygen with rate $\kappa_{\mathcal{P}_\gamma}$. Quiescent cells also consume oxygen but at a lower rate $\kappa_{\mathcal{Q}_\gamma} = 0.5 \times \kappa_{\mathcal{P}_\gamma}$.

Measurements of oxygen consumption by the PC3 Ctrl and PC3 Res cells cultured in monolayers revealed that the Ctrl cells consume more oxygen than Res cells by a factor of 1.4 (see main text). As

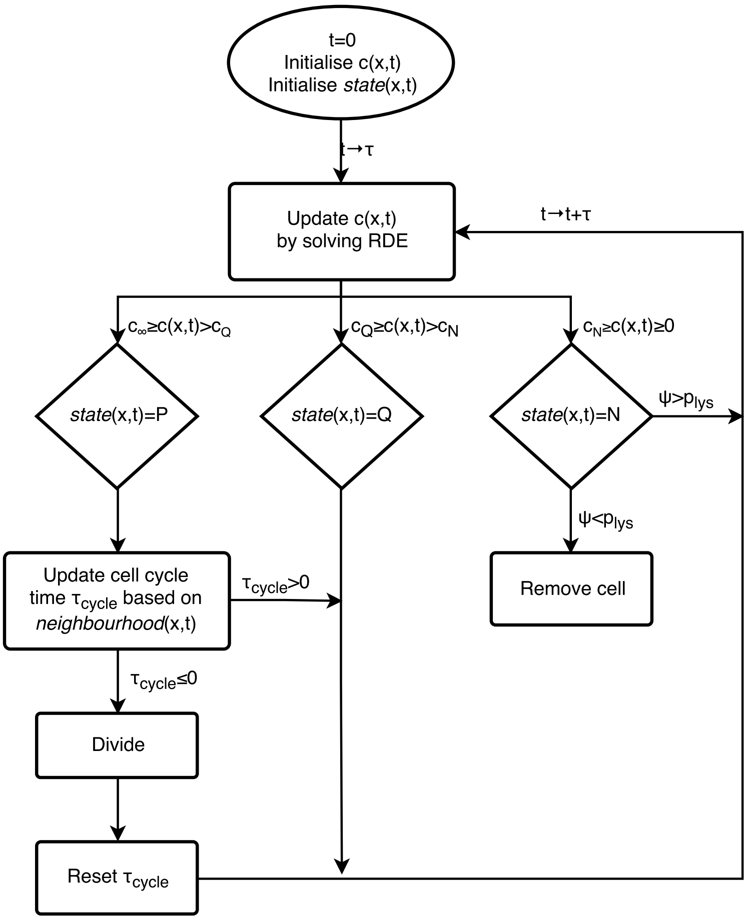

Figure 6: Flowchart summarising the algorithm used to implement the CA model.

shown previously, cells in tumour spheroids are known to consume less oxygen than in monolayers [4]. In the absence of estimates of the O_2 consumption rates in the PC3 spheroids, we treated $\kappa_{\mathcal{P}_\gamma}$ as free parameters and explored their influence on spheroid growth. Estimates of the values of $c_{\mathcal{Q}_\gamma}$ and $c_{\mathcal{N}_\gamma}$ were also difficult to obtain and so they were also treated as free parameters (see Sec.).

Cell cycle duration

We estimated cell cycle times for the PC3 Ctrl and PC3 Res cell populations by seeding a small number ($N_0 = N(t = 0) = 10^4$) of cells in a Petri dish, allowing them to expand for 48 hours and counting $N(t = 48)$, the number of viable cells at $t = 48$ hours. By assuming that the cells grew exponentially during this time period with growth rate k (so that $N(t) = N_0 e^{kt}$), we estimated the growth rate k (h^{-1}) from the data as follows,

$$k = \frac{\log\left(\frac{N_{48}}{N_0}\right)}{48}.$$

Given k, the doubling time t_d can be calculated via $t_d = \frac{\log(2)}{k}$ hours. We used t_d to estimate the cell cycle duration time. Thus the average cell cycle time of the PC3 Ctrl was estimated to be $\bar{\tau}_{cycle_{\mathcal{C}}} = 18.3$ h with standard deviation $\sigma_{cycle_{\mathcal{C}}} = 1.4$ h. The PC3 Res cells divided more rapidly, with mean $\bar{\tau}_{cycle_{\mathcal{R}}} = 16.9$ h and standard deviation $\sigma_{cycle_{\mathcal{R}}} = 0.9$ h. These estimates were based on six independent measurements.

Parameter inference

We used a two step process to estimate the values of parameters $c_{\mathcal{Q}_\gamma}$, $c_{\mathcal{N}_\gamma}$, $\kappa_{\mathcal{P}_\gamma}$ and p_{lys_γ} for the PC3 Ctrl and PC3 Res cells. First, we used our hybrid CA model to simulate the growth of homogeneous spheroids for a range of values of the model parameters. We then compared the simulation results to growth curves from the PC3 Ctrl and PC3 Res homogeneous spheroids and experimental images of their spatial distributions. In this way, we identified those values of $c_{\mathcal{Q}_\gamma}$, $c_{\mathcal{N}_\gamma}$, $\kappa_{\mathcal{P}_\gamma}$ and p_{lys_γ} that best fit the data. We then fixed these parameter values in the hybrid CA model when simulating the growth of heterogeneous spheroids.

In more detail, to identify the values of $c_{\mathcal{Q}_\gamma}$, $c_{\mathcal{N}_\gamma}$, $\kappa_{\mathcal{P}_\gamma}$ and p_{lys_γ} that best fit the growth curves for a given cell population $\gamma \in \{$PC3 Ctrl, PC3 Res$\}$ we ran the CA model for a single population γ for the following ranges of parameters: $c_{\mathcal{Q}_\gamma} \in (0, 2.8 \times 10^{-7})$, $c_{\mathcal{N}_\gamma} \in (0, c_{\mathcal{Q}_\gamma})$, $\kappa_{\mathcal{P}_\gamma} \in (1.0 \times 10^{-9}, 1.0 \times 10^{-8})$ and $p_{lys_\gamma} \in (0, 0.5)$. We compared the *in silico* growth curves to the experimental PC3 Ctrl and PC3 Res growth curves by calculating the sum of squared residuals between them and shortlisted ten sets of parameters that resulted in *in silico* growth curves with the lowest sum of squares for each population. We then compared the spatial distributions for the shortlisted *in silico* spheroids to the available images of homogeneous PC3

Table 2: Summary of the parameters used in the CA model together with estimates of their values.

**Parameter**	**Description**	**Value**	**Units**	**Ref.**
l_{PC3}	Cell size	0.0018	cm	[2]
L	Domain length	0.36	cm	Estimated
$\bar{\tau}_{cycle_C}(\sigma_{cycle_C})$	Mean (standard deviation) cell cycle time (PC3 Ctrl)	18.3 (1.4)	h	Estimated
$\bar{\tau}_{cycle_R}(\sigma_{cycle_R})$	Mean (standard deviation) cell cycle time (PC3 Res)	16.9 (0.9)	h	Estimated
c_∞	Background O_2 concentration	2.8×10^{-7}	mol cm^{-3}	[4]
D	O_2 diffusion constant	1.8×10^{-5}	cm^2 s^{-1}	[6]
c_{Q_C}	O_2 concentration threshold for Ctrl proliferating cells	1.82×10^{-7}	mol cm^{-3}	Estimated
c_{N_C}	O_2 concentration threshold for Ctrl quiescent cells	1.68×10^{-7}	mol cm^{-3}	Estimated
c_{Q_R}	O_2 concentration threshold for Res proliferating cells	2.24×10^{-7}	mol cm^{-3}	Estimated
c_{N_R}	O_2 concentration threshold for Res quiescent cells	2.1×10^{-7}	mol cm^{-3}	Estimated
κ_{P_C}	O_2 consumption rate of proliferating cells	1.0×10^{-8}	mol cm^{-3} s^{-1}	Estimated
κ_{P_R}	O_2 consumption rate of proliferating cells	3.3×10^{-9}	mol cm^{-3} s^{-1}	Estimated
κ_{Q_γ}	O_2 consumption rate of quiescent cells	$0.5\kappa_{P_\gamma}$	mol cm^{-3} s^{-1}	Estimated
p_{lys_C}	Rate of lysis of Ctrl cells	0.015	h^{-1}	Estimated
p_{lys_R}	Rate of lysis of Res cells	0.015	h^{-1}	Estimated

control and resistant spheroids and selected those sets of parameter values that best matched the images for both populations by visual inspection. The selected parameter sets for the control and resistant populations were subsequently used in the simulations of heterogeneous spheroids (see Table 2).

Fig. 7 shows typical results from simulations of our hybrid CA model. Fig. 7a shows how the spatial distribution of a homogeneous spheroid changes over time for a single realisation of the model whereas Fig. 7b represents the average cumulative volumes of the necrotic, quiescent and proliferating regions from 100 realisations of the model.

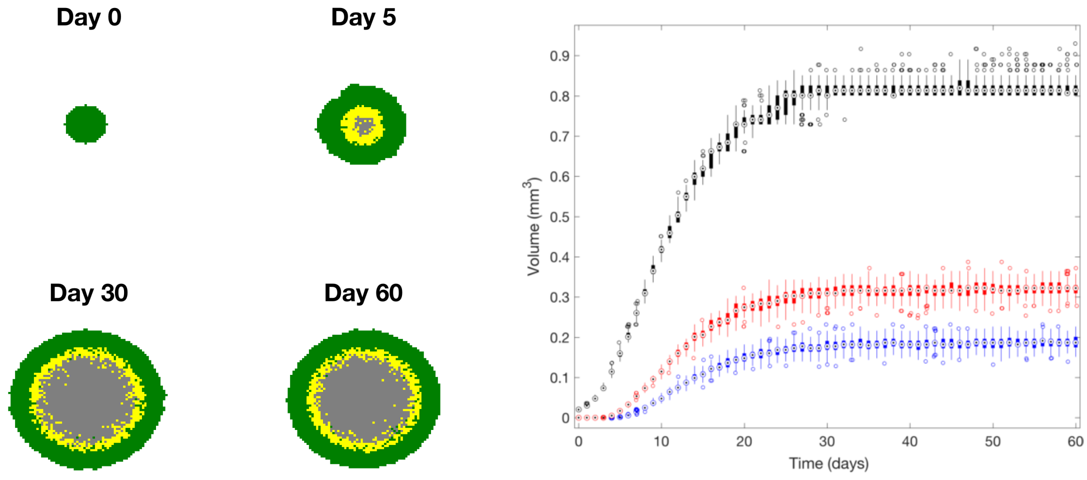

(a) **(b)**

Figure 7: Typical results from simulations of the 2D CA model showing (a) spatial distributions of spheroids on different days from a single realisation of the model and (b) growth curves obtained from 100 realisations. In (a), the proliferating cells are green, quiescent cells are yellow and necrotic cells are grey. Plot (b) shows the total volumes of spheroids (black), the volumes of necrotic regions (blue) and the combined volumes of the necrotic and quiescent regions (red). The circled dots represent the median values, the boxes represent the interquartile ranges and the whiskers extending from the boxes mark the min. and max. values. The empty circles represent outliers. Nondimensionalised parameter values used in the simulations: $\bar{\tau}_{cycle} = 18.3$, $\kappa_{\mathcal{P}} = 150$, $p_{lys} = 0.015$, $c_{\mathcal{Q}} = 0.8$ and $c_{\mathcal{N}} = 0.775$.

References

[1] Banks, H.T., Holm, K, and Robbins, D., *Standard error computations for uncertainty quantification in inverse problems: asymptotic theory vs. bootstrapping*, Math. Comput. Model., 52(9–10), 1610–1625, 2010.

[2] Nexcelom Bioscience, *http://www.nexcelom.com/landing/NCI-60-cell-table.php.*

[3] Coleman, T. F. and Li, Y., *An interior trust region approach for nonlinear minimization subject to bounds*, SIAM J. Optim., 6(2), 418–445, 1996.

[4] Freyer, J. P. and Tustanoff, E. and Franko, A. J. and Sutherland, R. M., *In situ oxygen consumption rates of cells in V-79 multicellular spheroids during growth*, J. Cell. Physiol., 118(1), 53–61, 1984.

[5] Gerlee, P., *The model muddle: In search of tumor growth laws*, Cancer Res., 73(8), 2407–2411, 2013.

[6] Grote, J. and Süsskind, R. and Vaupel, P., *Oxygen diffusivity in tumor tissue (DS-Carcinosarcoma) under temperature conditions within the range of 20–40° C*, Eur. J. Phycol., 372(1), 37–42, 1977.

[7] Jagiella, N. and Müller, B. and Müller, M. and Vignon-Clementel, I. E. and Drasdo, D., *Inferring growth control mechanisms in growing multi-cellular spheroids of NSCLC cells from spatial-temporal image data*, PLoS Comput. Biol., 12(2), e1004412, 2016.

[8] Jiang, Y. and Pjesivac-Grbovic, J. and Cantrell, C. and Freyer, J. P., *A multiscale model for avascular tumor growth*, Biophys. J., 89(6), 3884–3894, 2005.

[9] O'Rourke, S. and McAneney, H. and Hillen, T., *Linear quadratic and tumour control probability modelling in external beam radiotherapy*, J. Math. Biol., 58(4–5), 799–817, 2009.

[10] Tzedakis, G. and Tzamali, E. and Marias, K. and Sakkalis, V., *The importance of neighborhood scheme selection in agent-based tumor growth modeling*, Cancer Inform., 14(S4), 67–81, 2015.

Supplementary Methods 3: Statistical analysis

Cell cycle analysis

We set out to test the null hypothesis that the proportions of cells in G0G1, EdU+, and G2M phases do not change when resistant and control cells are cultured in homogeneous or mixed spheroids after five, ten, and 15 days of culture. We inspected cell distributions shown in Figure 1 and identified `expdate` as a potential confounding factor. For each combination of irradiation status (TRUE or FALSE), cell type (pc3 and du145), cell resistance status (resistant and control), and days after culturing (5, 10, and 15) we performed the following analysis. Due to overdispersion in the data, a Poisson regression model was not appropriate, and we instead used a negative-binomial model [6] to model cell counts (`counts`) under the alternative hypothesis using the model specification in Wilkinson-Rogers notation [7]:

```
counts ~ group + phase + expdate + group:phase
```

where `group`, `phase`, and `expdate` are main effects, and `group:phase` is an interaction effect between `group` and `phase`. `group` represents the type of culture (homogeneous or mixed), `phase` represents the cell-cycle phase (G0G1, EdU+, or G2m), and `expdate` represents the date each cell count experiment was performed. We generated half-normal plots of model fit residuals and simulated envelopes for these plots (data not shown) using the `hnp` package [3] in `R` [5]. The half-normal plots showed one model fit with unsatisfactory residual deviances, and this model was removed from further analysis. We tested the null hypothesis by testing if the inclusion of the interaction effect `group:phase` significantly reduces the deviance of the model using a Chi-square test as implemented in the `anova.negbin` function in the `MASS` package [6]. We corrected the 23 p-values obtained in this way for multiple testing with the Benjamini-Hochberg procedure [1]. We report six significant tests at a false discovery rate of 0.05 in Table 1.

Cell death rates

We next investigated whether the null hypothesis that the proportions of dead and alive cells did not vary between homogeneous and mixed spheroids. The cell counts shown in figure 2 indicate that these proportions vary at day 15 and to a lesser extent at day 10, and that experiment date may have an effect on death rates. Due to overdispersion in the data, we fit a quasibinomial model [6] with the specification

```
counts ~ group + expdate
```

using the `glm` function in `R`, where `group` and `expdate` are defined as in the previous analysis, and `counts` is a matrix of alive and dead cell counts. We generated half-normal plots with simulated envelopes as in the previous

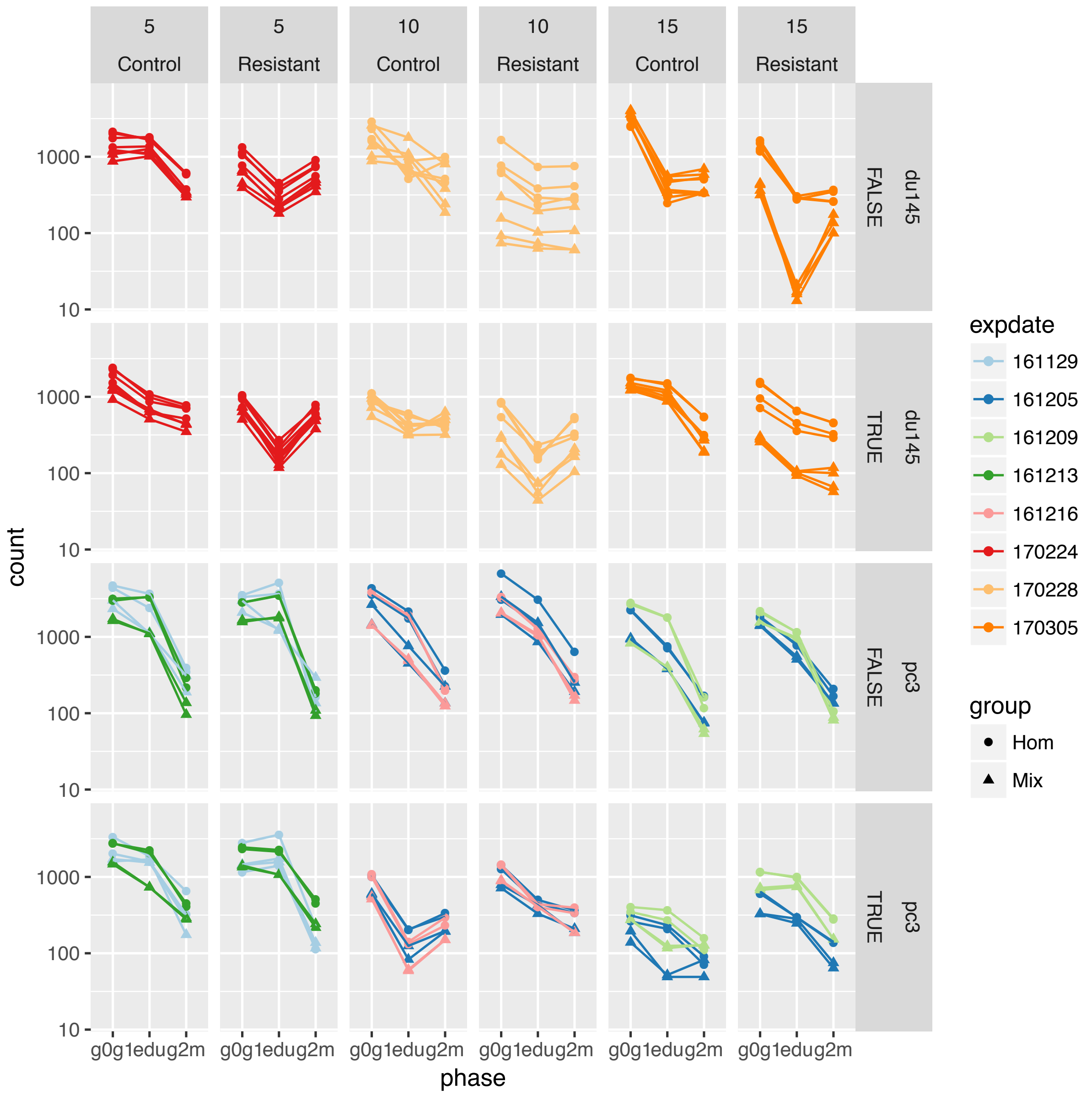

Figure 1: Cell counts by cell-cycle phase for each combination of days of culture, resistance status, cell type, and irradiation status. **expdate** is experiment date and **group** is the type of spheroid where Hom is homogeneous and Mix is mixed.

analysis (data not shown) and excluded two model fits due to poor fit on the basis of these plots. We tested the null hypothesis by testing if the inclusion of the `group` effect significantly reduces the deviance of the model using an F test as implemented in the `anova.glm` function in the `stats` package [5]. We correct the remaining 22 model fit p-values for multiple testing using the Benjamini-Hochberg procedure [1]. We report 9 significant tests at a false discovery rate of 0.05 in Table 2.

ir	cell_type	stain	day	p	HXg0g1	HXg2m	fold_change	BH	in_Hom
FALSE	pc3	gfp	5	0.0065	-0.49	-0.5	0.35	0.019	edu+ up
FALSE	pc3	gfp	10	5.6e-05	-0.43	-0.78	0.4	0.00032	edu+ up
FALSE	pc3	gfp	15	0.31	-0.13	-0.32	0.1	0.64	not significant
FALSE	pc3	dsred	5	0.0018	-0.52	-0.87	0.32	0.0071	edu+ up
FALSE	pc3	dsred	10	0.62	0.13	0.24	-0.094	0.8	not significant
FALSE	pc3	dsred	15	1	0.013	0.018	-0.0093	1	not significant
FALSE	du145	gfp	5	0.49	0.13	0.15	-0.072	0.8	not significant
FALSE	du145	gfp	10	0.0046	0.93	1.1	-0.5	0.015	edu+ down
FALSE	du145	gfp	15	0.77	-0.08	0.078	0.057	0.89	not significant
FALSE	du145	dsred	5	0.62	0.12	-0.036	-0.044	0.8	not significant
FALSE	du145	dsred	10	0.55	0.45	0.017	-0.19	0.8	not significant
FALSE	du145	dsred	15	3.1e-27	-1.6	-2	3.6	7.1e-26	edu+ up
TRUE	pc3	gfp	5	0.97	0.019	0.056	-0.015	1	not significant
TRUE	pc3	gfp	10	0.19	-0.11	-0.21	0.13	0.45	not significant
TRUE	pc3	gfp	15	9.8e-12	-0.75	-1.1	0.81	1.1e-10	edu+ up
TRUE	pc3	dsred	5	0.88	-0.11	0.052	0.047	0.96	not significant
TRUE	pc3	dsred	10	2.6e-10	0.39	0.49	-0.27	2e-09	edu+ down
TRUE	pc3	dsred	15	0.00014	0.38	0.45	-0.21	0.00065	edu+ down
TRUE	du145	gfp	5	0.55	0.16	0.12	-0.11	0.8	not significant
TRUE	du145	gfp	10	0.0089	-0.19	-0.53	0.26	0.023	edu+ up
TRUE	du145	gfp	15						
TRUE	du145	dsred	5	0.72	-0.04	-0.13	0.07	0.87	not significant
TRUE	du145	dsred	10	0.46	0.13	-0.17	-0.0079	0.8	not significant
TRUE	du145	dsred	15	0.61	-0.22	-0.15	0.17	0.8	not significant

Table 1: Results of cell-cycle model fits. **Column names**: **ir**: Whether cells were irradiated; **day**: Days after seeding; **p**: p-value of Chi-square test; **HXg0g1**: Effect size of Homogeneous:g0g1 interaction effect; **HXg2m**: Effect size of Homogeneous:g2m interaction effect; **fold_change**: Fold increase of proportion of EdU+ cells in homogeneous culture; **BH**: Benjamini-Hochberg adjusted p-value; **in_homogeneous**: Direction of EdU+ proportion change in homogeneous spheroids. Model parameters and p-values are not reported for models with a poor fit.

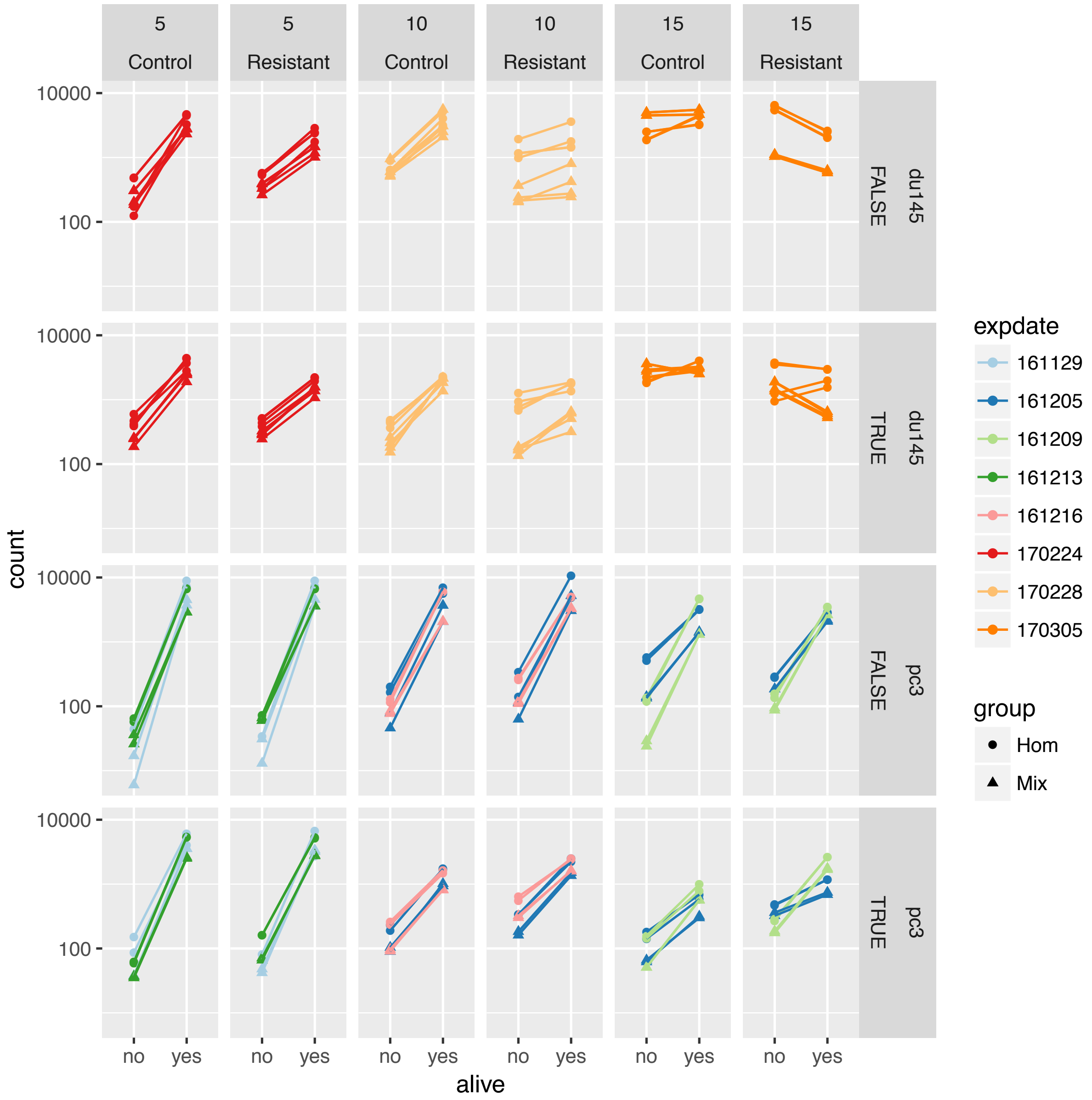

Figure 2: Number of dead and alive cells in each cell-cycle for each combination of days of culture, resistance status, cell type, and irradiation status. **expdate** is experiment date and **group** is the type of spheroid where Hom is homogeneous and Mix is mixed.

ir	cell_type	stain	day	p	BH	BH_sig	odds_ratio
FALSE	du145	dsred	10	0.76	0.76	FALSE	0.93
FALSE	du145	dsred	15				
FALSE	du145	dsred	5	0.011	0.045	TRUE	1.3
FALSE	du145	gfp	10	0.44	0.5	FALSE	1.1
FALSE	du145	gfp	15	0.017	0.045	TRUE	1.6
FALSE	du145	gfp	5	0.46	0.51	FALSE	1.3
FALSE	pc3	dsred	10	0.00011	0.0023	TRUE	0.67
FALSE	pc3	dsred	15	0.013	0.045	TRUE	0.86
FALSE	pc3	dsred	5	0.14	0.22	FALSE	1.4
FALSE	pc3	gfp	10	0.52	0.54	FALSE	1.2
FALSE	pc3	gfp	15	0.0051	0.038	TRUE	0.59
FALSE	pc3	gfp	5	0.37	0.45	FALSE	0.82
TRUE	du145	dsred	10	0.11	0.19	FALSE	0.58
TRUE	du145	dsred	15	0.0052	0.038	TRUE	2.8
TRUE	du145	dsred	5	0.11	0.19	FALSE	0.88
TRUE	du145	gfp	10	0.015	0.045	TRUE	0.54
TRUE	du145	gfp	15				
TRUE	du145	gfp	5	0.3	0.39	FALSE	0.8
TRUE	pc3	dsred	10	0.025	0.054	FALSE	0.81
TRUE	pc3	dsred	15	0.11	0.19	FALSE	1.1
TRUE	pc3	dsred	5	0.21	0.29	FALSE	0.83
TRUE	pc3	gfp	10	0.018	0.045	TRUE	0.76
TRUE	pc3	gfp	15	0.0093	0.045	TRUE	0.66
TRUE	pc3	gfp	5	0.18	0.26	FALSE	0.67

Table 2: Results of cell-death model fits. Column names: **ir**: Whether cells were irradiated; **day**: Days after seeding; **p**: p-value of F test; **BH**: Benjamini-Hochberg adjusted p-value; **BH_sig**: Whether the adjust p-value is significant at a false discovery rate of 0.05; **odds_ratio**: Fold increase in chance of cell being dead when isolated from homogeneous spheroids compared to mixed spheroids. Model parameters and p-values are not reported for models with a poor fit

Post-radiation growth experiments

Volumes for spheroids irradiated on day 4 after seeding were available for PC3 and DU145 spheroids seeded from several mixtures of radio-resistant and radio-sensitive cells irradiated between 2.5 Gy and 20 Gy (See Fig 4 in main text), as well as for unirradiated cells. All spheroid volumes were log_{10} transformed. Logarithmic spheroid increase was calculated for each sampling date after irradiation by subtracting the log_{10} volume on day 4 from the log_{10} volume measured on that day. Baseline growth delay for each cell mixture and experiment was estimated as the time point at which unirradiated spheroids crossed a cutoff growth increase (2.5x, 3x, and 4x for data presented in Fig 4a, b, and e, respectively) by fitting a local polynomial regression [2] (exact method) and estimating the fit's intersection with the cutoff line through bisection [4]. Time until regrowth to the cutoff volume was estimated as the first sampling date on which a spheroid had a larger growth increase than the cutoff. Growth delay for irradiated spheroids was estimated as the time until regrowth minus the baseline growth delay. Irradiated spheroids with a negative growth delay were excluded from the analysis. Spheroids that did not regrow past the regrowth cutoff were censored.

Co-culture transwell experiments

We analyzed cell counts from a series of co-culture experiments of resistant and control cells in hypoxia and normoxia on six different days. Each experiment was performed in triplicate. Figure 3 shows that in hypoxia, counts in the non control/control groups (`C/R`, `R/C`, and `R/R`) may be elevated compared to the control/control co-culture (`C/C`). To establish whether cell counts were elevated in non-`C/C` co-culture in hypoxia, and whether cell counts did not differ between co-culture types in normoxia, we modelled cell counts (`count`) as depending on the day of the experiment (`expdate`) and the type of co-culture (`group`) using the model specification

```
counts ~ group*condition + expdate
```

Due to overdispersion in the data, we fit this model using a negative-binomial model. Half-normal plots showed a good fit of the model to the data (data not shown). We used the `R` function `summary.negbin` from the `MASS` package [6] to perform a Wald test on the `group` effects of the model. In normoxia, no culture type had a statistically significant difference in counts compared to `C/C`. In hypoxia, all three culture types differed from `C/C` (`C/R`: p=0.0038, z=3.54; `R/R`: p=8.7e-05, z=4.23; `R/C`: p=0.0036, z=2.86).

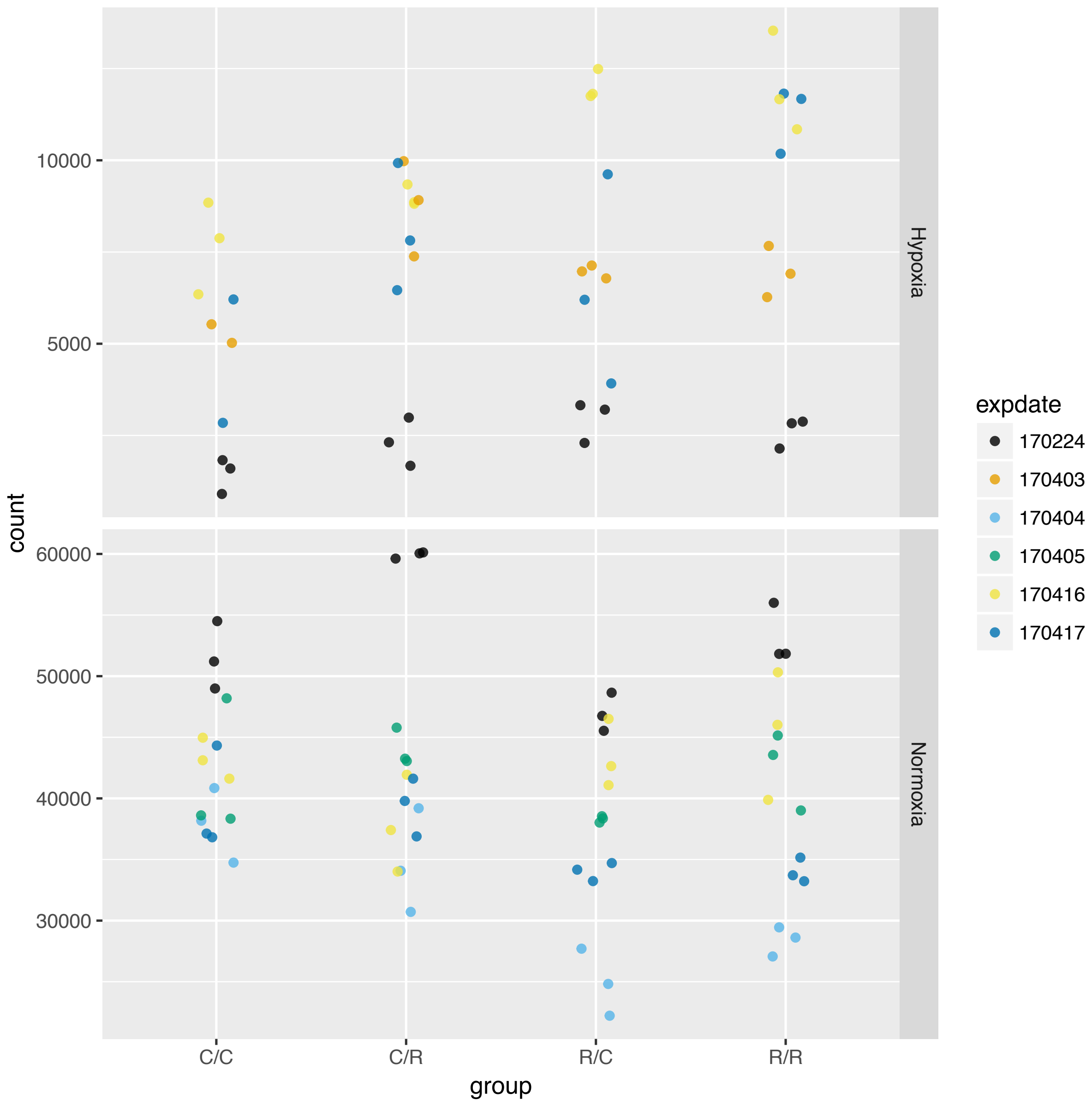

Figure 3: Cell counts of co-culture experiments. Each point represents a technical replicate. Each color represents an experiment date. In hypoxia, cell counts are somewhat higher in C/R, R/C, and R/R compared to C/C.

References

[1] Y Benjamini and Y Hochberg. Controlling the False Discovery Rate: A Practical and Powerful Approach to Multiple Testing. *J. R. Stat. Soc. Ser. B*, 57(1):289–300, 1995.

[2] WS Cleveland, E Grosse, and WM Shyu. *Local regression models. Chapter 8 in Statistical models in S (JM Chambers and TJ Hastie eds.), 608 p.* Wadsworth & Brooks/Cole, Pacific Grove, CA, 1992.

[3] RA Moral, J Hinde, and CGB Demétrio. Half-normal plots and overdispersed models in r: The hnp package. *J. Stat. Softw.*, 81:23, 2017.

[4] Alfio Quarteroni, Riccardo Sacco, and Fausto Saleri. *Numerical mathematics, second edition*. Springer-Verlag, Berlin, 2007.

[5] R Core Team. *R: A Language and Environment for Statistical Computing*. R Foundation for Statistical Computing, Vienna, Austria, 2018.

[6] WN Venables and BD Ripley. *Modern applied statistics with S-Plus*. Springer, 2002.

[7] GN Wilkinson and CE Rogers. Symbolic description of factorial models for analysis of variance. *J. R. Stat. Soc. Ser. C*, 22(3):392–399, 1973.
