# Supplementary material for: Reciprocal interactions between tumour cell populations enhance growth and reduce radiation sensitivity in prostate cancer
Source: Commun Biol. 2021 Jan 4;4:6. doi: 10.1038/s42003-020-01529-5 (PMC7782740; doi:10.1038/s42003-020-01529-5)
Supplement: Supplementary file 2 — Reporting Summary [file 42003_2020_1529_MOESM2_ESM.pdf]

nature research

Corresponding author(s): Pavitra Kannan

Last updated by author(s): Oct 25, 2020

Reporting Summary

Nature Research wishes to improve the reproducibility of the work that we publish. This form provides structure for consistency and transparency in reporting. For further information on Nature Research policies, see our Editorial Policies and the Editorial Policy Checklist.

Statistics

For all statistical analyses, confirm that the following items are present in the figure legend, table legend, main text, or Methods section.

n/a	Confirmed	
☐	☒	The exact sample size (*n*) for each experimental group/condition, given as a discrete number and unit of measurement
☐	☒	A statement on whether measurements were taken from distinct samples or whether the same sample was measured repeatedly
☐	☒	The statistical test(s) used AND whether they are one- or two-sided<br>*Only common tests should be described solely by name; describe more complex techniques in the Methods section.*
☒	☐	A description of all covariates tested
☐	☒	A description of any assumptions or corrections, such as tests of normality and adjustment for multiple comparisons
☐	☒	A full description of the statistical parameters including central tendency (e.g. means) or other basic estimates (e.g. regression coefficient) AND variation (e.g. standard deviation) or associated estimates of uncertainty (e.g. confidence intervals)
☐	☒	For null hypothesis testing, the test statistic (e.g. *F*, *t*, *r*) with confidence intervals, effect sizes, degrees of freedom and *P* value noted<br>*Give P values as exact values whenever suitable.*
☒	☐	For Bayesian analysis, information on the choice of priors and Markov chain Monte Carlo settings
☒	☐	For hierarchical and complex designs, identification of the appropriate level for tests and full reporting of outcomes
☒	☐	Estimates of effect sizes (e.g. Cohen's *d*, Pearson's *r*), indicating how they were calculated

Our web collection on statistics for biologists contains articles on many of the points above.

Software and code

Policy information about availability of computer code

Data collection	Celigo Cytometer (v 3.1), GelCount (Oxford Optronix), and Incucyte (BioEssen) were used to image cell colonies in clonogenic assays, and to count cell numbers for growth and Transwell assays. Nikon TiE with NIS Elements Advanced Research software (16-bit) was used to acquire epifluorescence images of spheroid sections. Aperio CS2 slide scanner (Leica) was used to acquire H/E images. Seahorse XF Analyzer was used to acquire OCR measurements. Custom-code written in Matlab (v. 2018) was used for cellular automaton simulations.
Data analysis	Matlab (v 2018) was used for mathematical modelling and for calculation of spheroid volumes (Spheroid Sizer). FlowJo (v 10) was used to analyse flow cytometry data. GraphPad Prism (v 8.0) was used for statistical analysis of all biological experiments, except for ones with heteroscedasticity, for which R (v 3.4) project was used.

For manuscripts utilizing custom algorithms or software that are central to the research but not yet described in published literature, software must be made available to editors and reviewers. We strongly encourage code deposition in a community repository (e.g. GitHub). See the Nature Research guidelines for submitting code & software for further information.

Data

Policy information about availability of data

All manuscripts must include a data availability statement. This statement should provide the following information, where applicable:

- Accession codes, unique identifiers, or web links for publicly available datasets
- A list of figures that have associated raw data
- A description of any restrictions on data availability

Data associated with all the main and supplementary figures in this study are publicly available in Zenodo with DOI 10.5281/zenodo.4130692.

Field-specific reporting

Please select the one below that is the best fit for your research. If you are not sure, read the appropriate sections before making your selection.

- [x] Life sciences
- [ ] Behavioural & social sciences
- [ ] Ecological, evolutionary & environmental sciences

For a reference copy of the document with all sections, see nature.com/documents/nr-reporting-summary-flat.pdf

Life sciences study design

All studies must disclose on these points even when the disclosure is negative.

Sample size	For in vitro experiments, no sample size calculation was performed prior to experiment. However, sample sizes were approximated using effect sizes from pilot studies to ensure power (approximate beta value of 0.8).
Data exclusions	Fig 1: Data are missing from some time points due to technical failures in imaging.<br>Fig 2: one DU145 mixed spheroid was excluded from analysis, as it was an outlier in terms of growth compared to all other 35 spheroids.<br>Fig 4: one plate of PC3 spheroids was excluded from analysis, as the growth of these spheroids did not resemble the growth of spheroids in the 9 other plates.
Replication	At least two separate batches of cells were used for experimental replicates. Findings were reproduced upon replication, except for the data mentioned above (likely due to experimental error).
Randomization	For in vitro experiments, randomization was not possible. However, experiments were set up in a way to ensure that all experimental groups (parental, mix, radres) were seeded on the same plate to avoid plate-plate variability.
Blinding	For in vitro experiments, blinding was not possible for data collection. However, when possible, analysis was performed using automated image analysis to minimize bias.

Reporting for specific materials, systems and methods

We require information from authors about some types of materials, experimental systems and methods used in many studies. Here, indicate whether each material, system or method listed is relevant to your study. If you are not sure if a list item applies to your research, read the appropriate section before selecting a response.

Materials & experimental systems

n/a	Involved in the study	
[ ]	[x]	Antibodies
[ ]	[x]	Eukaryotic cell lines
[x]	[ ]	Palaeontology and archaeology
[x]	[ ]	Animals and other organisms
[x]	[ ]	Human research participants
[x]	[ ]	Clinical data
[x]	[ ]	Dual use research of concern

Methods

n/a	Involved in the study	
[x]	[ ]	ChIP-seq
[ ]	[x]	Flow cytometry
[x]	[ ]	MRI-based neuroimaging

Antibodies

Antibodies used	For flow cytometry experiments, antibodies were: efluor 780 fixabile viability dye (65-0865-14, ThermoFisher); Click-iT EdU Alexa Fluor 647 (C10635, ThermoFisher); FxCycle Violet (F10347, ThermoFisher). For immunofluorescence experiments, antibodies included: Ki67 (VP-RM04,clone SP6, Vector); anti-EF5 antibody (ELK3-51 Cy5, University of Pennsylvania)
Validation	For flow experiments, antibodies were tested using positive controls and single stained samples. Live/dead markers were tested by mixing 50% dead cells (heated to 56 C, 5 min) and 50% live cells together prior to staining. For immunofluorescence images, an IgG-specific isotype control or a competed stain (EF5) was used to determine non-specific staining.

Eukaryotic cell lines

Policy information about cell lines

Cell line source(s)	Prostate cells were sourced from the Liu lab (University of Toronto).
Authentication	Prostate cell lines were authenticated using STR analysis.
Mycoplasma contamination	Cell lines were checked routinely for mycoplasma and tested negative using MycoAlert Mycoplasma Detection Kit (Lonza).

Commonly misidentified lines (See ICLAC register)	None

Flow Cytometry

Plots

Confirm that:

- [x] The axis labels state the marker and fluorochrome used (e.g. CD4-FITC).
- [x] The axis scales are clearly visible. Include numbers along axes only for bottom left plot of group (a 'group' is an analysis of identical markers).
- [x] All plots are contour plots with outliers or pseudocolor plots.
- [x] A numerical value for number of cells or percentage (with statistics) is provided.

Methodology

Sample preparation	Spheroids were incubated with EdU (10 µM final concentration) 12 h, dissociated using 100 µL Accumax for 20 minutes at 37 °C, washed with PBS, centrifuged at 300xg for 5 min, incubated with efluor-780 (1 µL/mL PBS) for 30 min on ice in the dark to distinguish live/dead cells, washed in PBS, and fixed for 10 min in IC Fixation Buffer. Cells were then permeabilized and stained with Click-iT EdU Alexa Fluor 647 according to manufacturer's instructions. After washing in 1X saponin, cells were incubated 30 min with FxCycle Violet Stain (1:1000 stock diluted in 300 µL of 1X saponin) before being measured.
Instrument	BD LSR Fortessa X-20 ; Attune NxT Flow Cytometer
Software	Data were analysed using FlowJo (v 10; Treestar, Inc).
Cell population abundance	For all samples, at least 10,000 events were collected.
Gating strategy	After excluding debris (SSC v FSC), doublets (FSC-H vs FSC-A), and dead cells (Cy7 vs FSC), parental (GFP+, Alexa Fluor 488) and radioresistant (DsRed+, PE-Texas Red) cells were analysed for cell cycle proportions. The uptake of the nucleoside analogue 5-ethynyl-2 deoxyuridine (EdU, Alexa Fluor 647) was detected both in cells proliferating in S phase and in cells that previously took up EdU in S phase and then cycled into G0G1 (Pacific Blue) during the incubation time. Gating was thus performed to include all proliferation (EdU+ cells) to account for turnover. Single stained and unstained samples were used to set up compensation and define positive vs negative staining.

- [x] Tick this box to confirm that a figure exemplifying the gating strategy is provided in the Supplementary Information.
